# Equity, Diversity, and Inclusion Concerns From JAMA Network Peer Reviewers

**DOI:** 10.1001/jamanetworkopen.2025.39886

**Published:** 2025-10-28

**Authors:** Michael O. Mensah, Anand R. Habib, Jacob Kendall-Taylor, Mya L. Roberson, Kanade Shinkai, Annette Flanagin, Preeti N. Malani

**Affiliations:** 1 Department of Psychiatry, Yale School of Medicine, New Haven, Connecticut; 2 Assistant Editor, *JAMA Network Open*, Chicago, Illinois; 3 Department of Medicine, University of California, San Francisco; 4 Director of Editorial Systems, *JAMA* and the JAMA Network, Chicago, Illinois; 5 Department of Health Policy and Management, University of North Carolina Gillings School of Global Public Health, Chapel Hill,; 6 Associate Editor, *JAMA Dermatology*, Chicago, Illinois; 7 Department of Dermatology, University of California, San Francisco; 8 Editor-in-Chief, *JAMA Dermatology*, Chicago, Illinois; 9 Managing Executive Editor, *JAMA*, Chicago, Illinois; 10 Department of Medicine, University of Michigan, Ann Arbor, Michigan; 11 Deputy Editor, *JAMA*, Chicago, Illinois

## Abstract

This cross-sectional study evaluates JAMA Network peer reviewers’ use of the equity, diversity, and inclusion concerns checkbox and used *JAMA Dermatology* as a case study to categorize concerns described in confidential comments.

## Introduction

Engaging with equity, diversity, and inclusion (EDI) issues in scientific peer review can promote more innovative research.^1^ Conversely, neglecting EDI issues during peer review has permitted publication of prejudicial articles that prompt retraction.^2^ Prior research^3^ suggests that EDI concerns in manuscripts garner more editorial attention when peer reviewers can flag those issues before publication. Beginning in March 2023, JAMA Network journals invited peer reviewers to check a box when manuscripts prompted EDI concerns and to explain such concerns in confidential comments to the editor. We examined JAMA Network peer reviewers’ use of the EDI concerns checkbox and used *JAMA Dermatology* as a case study to categorize concerns described in confidential comments.

## Methods

In this cross-sectional, mixed-methods analysis, we collated all manuscripts reviewed by the 13 JAMA Network journals between March 28, 2023, and December 31, 2024. For each journal, counts and percentages of peer reviews for which the EDI concerns checkbox had been marked yes were determined. For more detailed analysis, we then extracted the deidentified, confidential reviewer comments for *JAMA Dermatology* manuscripts and used rigorous and accelerated data reduction (RADAR) to analyze the comments.^4^ RADAR develops all-inclusive data tables, then iteratively reduces those tables until only research-question relevant data remain.^4^ Two racially minoritized former *JAMA* editorial fellows (M.O.M. and A.R.H.) independently classified confidential comments as either relevant or irrelevant to EDI, coded comments similarly to another study,^3^ agreed on which code(s) best described each comment, and then distilled prominent categories across all comments. This study was deemed exempt from review and the need for informed consent due to the use of deidentified data by the Yale Medical School institutional review board and follows Strengthening the Reporting of Observational Studies in Epidemiology (STROBE) and Standards for Reporting Qualitative Research (SRQR) reporting guidelines.

## Results

Among 39 151 manuscript reviews across all JAMA Network journals, 2075 (5.3%) used the EDI concerns checkbox, including 1897 of the 34 542 research manuscript reviews (5.5%) (Table 1). *JAMA Otolaryngology* reviews had the lowest proportion using the EDI checkbox (29 of 1104 [2.6%] overall; 26 of 948 [2.7%] research manuscripts). *JAMA Pediatrics* reviews had the highest proportion using the EDI checkbox (146 of 1793 [8.1%] overall; 126 of 1583 [8.0%] research manuscripts). *JAMA Dermatology* reviews used the EDI checkbox at the median frequency among all JAMA Network journals (91 of 1879 [4.8%] overall; 78 of 1439 [5.4%] research manuscripts).

**Table 1.** Proportion of Manuscript Peer Reviews Across JAMA Network Journals With a Reviewer-Identified Equity, Diversity, and Inclusion (EDI) Concern

Journal	Total manuscript peer reviews, No.	No. (%) of manuscript peer reviews utilizing the EDI checkbox, No. (%)
*JAMA*	8048	370 (4.60)
*JAMA Cardiology*	1691	71 (4.20)
*JAMA Dermatology*	1879	91 (4.84)
*JAMA Health Forum*	1352	68 (5.03)
*JAMA Internal Medicine*	1645	74 (4.50)
*JAMA Network Open*	12 766	829 (6.49)
*JAMA Neurology*	1896	94 (4.96)
*JAMA Oncology*	1521	78 (5.13)
*JAMA Ophthalmology*	1064	29 (2.73)
*JAMA Otolaryngology–Head and Neck Surgery*	1104	29 (2.63)
*JAMA Pediatrics*	1793	146 (8.14)
*JAMA Psychiatry*	1406	98 (6.97)
*JAMA Surgery*	2986	98 (3.28)
Median (IQR)	1691 (1406-1896)	91 [71-98] (4.84 [4.19-5.13])
Overall	39 151	2075 (5.30)

Among 91 *JAMA Dermatology* reviews using the EDI checkbox, 31 (34.1%) had confidential reviewer comments deemed relevant to EDI, 46 comments (50.5%) were assessed to be irrelevant to EDI, and 14 (15.4%) included no confidential comments. Regarding EDI-relevant confidential comments, categories of responses included: lack of clarity regarding race and ethnicity categories (15 comments [48.4%]), statistical or methodological concerns (5 comments [16.1%]), skin-tone representation (3 comments [9.7%]), ambiguity in language around demographic identifiers (3 comments [9.7%]), and inappropriate use of gender ascribed by observers (2 comments [6.5%]; Table 2).

**Table 2.** Representative, Deidentified, Equity, Diversity, and Inclusion–Relevant Confidential Reviewer Comments by Category and Prevalence

Category of concern	Prevalence, No. (%)^a^	Representative reviewer quote
Lack of clarity regarding race and ethnicity categories	15 (48.4)	“The rationale of race/ethnicity categorizations is not explained and hard to understand. The authors should categorize race/ethnicity in at least 4 categories…In the way that is done, it may seem that the categorization chosen was done because it affected the statistical significance.”
Statistical or methodological concerns	5 (16.1)	“A post hoc analysis like this…[seems like an attempt to address] interest in under-represented minorities in dermatology clinical trials, without actually addressing or trying to understand why we want to study those populations.”
Absence of diverse skin-tone representation	3 (9.7)	“As we grow our understanding of this disease…we should be mindful of ensuring appropriate representation. As such, the figure should include a presentation in skin of color if possible.”
Ambiguity in language around demographic identifiers	3 (9.7)	“More inclusive language suggested: assigned female at birth for girls; assigned male at birth for boys.”
Inappropriate use of gender ascribed by observers	2 (6.5)	“I recognize that self-identified gender, race, ethnicity data will not be available and yet these types of studies should be performed. We also make reflexive conscious and unconscious assessments of someone’s identity all the time and those assessments are influenced by our own biases. Maybe after lengthy discussion, we might conclude that the authors’ approach is reasonable and we would proceed through a consensus approach, being sure to include a thoughtful discussion on the rationale in the paper. It’s most troublesome to me that recognition of the authors’ own biases is not discussed anywhere in the paper.”

^a^ The total number of equity, diversity, and inclusion–relevant confidential comments does not sum to 31 (as reported in the manuscript text) as we have only included prevalence numbers as well as representative reviewer quotes in the table for those categories for which we coded at least 2 reviewer comments of that type.

## Discussion

Overall, manuscript peer reviewers across the JAMA Network journals infrequently used the EDI concerns checkbox. Among *JAMA Dermatology* reviews using the EDI concerns checkbox, less than half included confidential comments relevant to EDI. Consistent with EDI issues previously noted by the American Academy of Dermatology,^5^ prominent categories among such comments included concerns about proper usage of race and ethnicity categories, absence of photos depicting dermatologic conditions in darker skin tones, and use of inclusive language regarding sex and gender. Other medical journals might use EDI checkboxes to identify, quantify, and track EDI concerns about submitted manuscripts.

Our analysis has limitations. We cannot comment on how accurately checked EDI boxes reflected actual EDI issues in manuscripts, on EDI concerns in manuscripts that were not flagged, or on flagged manuscripts lacking comments to the editor. Future work could explore additional ways of soliciting EDI concerns from reviewers and of addressing such concerns before publication.

## Notes

Supplement. Data Sharing Statement
