# Supplementary material for: Equity, Diversity, and Inclusion Concerns From JAMA Network Peer Reviewers
Source: JAMA Netw Open. 2025 Oct 28;8(10):e2539886. doi: 10.1001/jamanetworkopen.2025.39886 (PMC12569707; doi:10.1001/jamanetworkopen.2025.39886)
Supplement: Supplement. — Data Sharing Statement [file jamanetwopen-e2539886-s001.pdf]

Data Sharing Statement

Mensah. Equity, Diversity, and Inclusion Concerns from JAMA Network Peer Reviewers. *JAMA Netw Open*. Published October 28, 2025. doi:10.1001/jamanetworkopen.2025.39886

Data
Data available: No

Additional Information
Explanation for why data not available: The data are confidential. A decision to make the data available in some form would have to be approved by JAMA leadership.
